# Metabolomics Approach Explore Diagnostic Biomarkers and Metabolic Changes in Heat-Stressed Dairy Cows

**DOI:** 10.3390/ani10101741

**Published:** 2020-09-25

**Authors:** Shuangming Yue, Siyan Ding, Jia Zhou, Chao Yang, Xiaofei Hu, Xiaonan Zhao, Zhisheng Wang, Lizhi Wang, Quanhui Peng, Bai Xue

**Affiliations:** 1 Animal Nutrition Institute, Sichuan Agricultural University, Chengdu 611130, China; yueshuangming@hotmail.com (S.Y.); dalanmaoding0906@sina.com (S.D.); zhoujia1@stu.sicau.edu.cn (J.Z.); hxf8982@126.com (X.H.); zxn780213675@126.com (X.Z.); wangzs@sicau.edu.cn (Z.W.); 12825@sicau.edu.cn (L.W.); 14101@sicau.edu.cn (Q.P.); 2 Department of Bioengineering, Sichuan Water Conservancy Vocation College, Chengdu 611845, China; mxp886@sicau.edu.cn

**Keywords:** heat stress, dairy cow, milk production, metabolomics, biomarkers

## Abstract

**Simple Summary:**

Heat stress results in a decline in the production performance of the dairy cows. This study explored the metabolomes of milk and blood plasma in heat-stressed cows by means of ^1^H nuclear magnetic resonance (^1^H NMR)-based metabolomics tools. Findings of the current experiment reveal that HS alters the metabolic composition of milk and blood plasma in lactating dairy cows. In brief, HS alters twelve metabolites in blood plasma and eight metabolites in milk, which are primarily involved in proteolysis, gluconeogenesis, and milk fatty acid synthesis, suggesting that these metabolites could be possible biomarkers for heat-stressed dairy cows.

**Abstract:**

In the present experiment, we investigated the impact of heat stress (HS) on physiological parameters, dry matter intake, milk production, the metabolome of milk, and blood plasma in lactating Holstein dairy cows. For this purpose, 20 Holstein lactating cows were distributed in two groups in such a way that each group had 10 cows. A group of 10 cows was reared in HS conditions, while the other group of 10 cows was reared in the thermoneutral zone. The results of the experiment showed that cows subjected to HS had higher respiration rates (*p* < 0.01) and greater rectal temperature (*p* < 0.01). Results of milk production and composition explored that HS lowered milk production (*p* < 0.01) and milk protein percentage (*p* < 0.05) than cows raised in a thermoneutral place. Furthermore, HS increased the concentrations of N-acetyl glycoprotein, scyllo-inositol, choline, and pyridoxamine in milk, while HS decreased the concentrations of O-acetyl glycoprotein, glycerophosphorylcholine, citrate, and methyl phosphate in milk. Moreover, HS enhanced plasma concentrations of alanine, glucose, glutamate, urea, 1-methylhistidine, histidine, and formate in cows, while the plasma concentration of low-density lipoprotein, very-low-density lipoprotein, leucine, lipid, and 3-hydroxybutyrate decreased due to HS. Based on the findings of the current research, it is concluded that HS alters the milk and blood plasma metabolites of lactating Holstein dairy cows. Overall, in the current experiment, HS altered eight metabolites in milk and twelve metabolites in the plasma of lactating Holstein dairy cows. Furthermore, the current study explored that these metabolites were mainly involved in proteolysis, gluconeogenesis, and milk fatty acid synthesis and could be potential biomarkers for dairy cows undergoing HS.

## 1. Introduction

Heat stress (HS) is negatively influencing animal health, production, and reproduction performance in modern livestock production systems [1]. The impact of HS on animals’ health, production, and reproduction performance is variable in different livestock species and breeds [2,3]. In beef cattle, HS is generally considered less crucial than dairy cattle because beef cattle have lower body size and metabolic rate, which results in lower body heat production [2,3]. Hence, dairy researchers are interested in conducting research on a topic related to the effect of HS on dairy animals’ health, production, and reproduction performance. Holstein dairy cow is a popular dairy breed among dairy farmers due to its high milk production. However, the potential of Holstein cows to emit body heat by skin evaporation is reduced in hot and humid environments [4]. Therefore, it is assumed that Holstein cows are at a greater risk of facing HS [5]. Findings of previous experiments have explored that HS in dairy cows not only reduces milk production and milk quality but also results in metabolic disorders, rumen acidosis, negative energy balance, and even death [6,7,8]. These metabolic disorders and health problems are directly linked with economic losses in the dairy industry [2,9,10]. 

In dairy cow’s production systems, the temperature and humidity index (THI) is considered as an index of the HS. Nevertheless, THI is only based on environmental humidity and temperature [1] and is not a direct biomarker of metabolic changes in dairy cows in response to HS. Therefore, in the dairy production system, vigorous metabolite biomarkers are required to identify the HS occurrence and to provide understandings related to physiological mechanisms in heat-stressed dairy cows.

Previous studies’ findings have explained that HS in lactating dairy cows not only results in reduced milk production but also negatively influences the milk protein contents [11,12]. It is generally considered that lower dry matter intake (DMI) is the major reason for reduced milk production and milk protein contents in heat-stressed dairy cows [7,13]. However, the latest studies have reported that lower dry matter intake partially (about 35–50%) describes the reduction in milk production and milk composition in dairy cows [11,12]. Therefore, it could be assumed that change in post-absorptive metabolism may be responsible for a large portion of the reduction in productivity of dairy cows; however, the mechanisms triggering these changes still remain vague [14].

Metabolomics, a useful tool for analyzing the changes of metabolites in physiological fluids and tissues in response to internal and external stimulations, has been successfully applied for filtering out biomarkers for milk quality [15], energy metabolism [16], and rumen health in dairy cows [14]. However, only a few studies have attempted to identify biomarkers for the diagnosis of HS in dairy cows based on milk [15] and plasma [16] metabolomics. Previous studies have reported that blood and milk metabolites of lactating dairy cows change during HS [14,17,18,19]. However, to date, none of the studies have been carried out to evaluate the effect of HS on both milk and plasma metabolome and their association on the milk yield of mid-lactating dairy cows. In this study, we formulated the hypothesis that dairy cows suffering from HS may manifest signs of milk and blood metabolite changes, which can be used as biomarkers for evaluating the influence of HS on dairy cows. Furthermore, the other objective of the current experiment was to find changes in metabolic pathways due to HS and to identify the relationship of metabolic changes with milk yield reduction in heat-stressed dairy cows by comparing the metabolomes of milk and plasma between cows reared in HS and thermoneutral (TN) conditions.

## 2. Materials and Methods

The current experimental trial was conducted at Mei Jiadun Dairy Farms (Huang Gang, Hubei, China). All experimental procedure was in accordance with Chinese laws on Animal Experimentation (GB/T 35892-2018). Furthermore, all research protocols were permitted by the Institute Animal Care Committee of Sichuan Agricultural University, China (#SCAUAC201408-3).

### 2.1. Experimental Design and Animal Management

A total of twenty healthy Holstein cows from the Mei Jiadun Dairy Farms (Huang Gang, Hubei, China) herd, with healthy and symmetrical udders, were selected for the current experiment. All cows were similar in parity, days in milk, milk yield, and body weight (Table 1). Cows were maintained and reared under the same feeding and management regime before the start of the experiment. For these experiments, experimental animals were kept in a cowshed (closed type) to avoid environmental variation and photoperiods on cow’s metabolism. Shed (150 m × 20 m) was equally divided into 10 parts with the help of temporary sidewalls of bamboos and steel pipes. Each part was designated to a single cow. Every part of the barn was bedded with geotextile mattresses and topped with a thin layer of sawdust. Each part of the barn contained a drinker and fan. To avoid light, shading nets were used around the cowshed.

Each experimental animal was reared in an individual pen. Fresh drinking water was ensured for each pen. All other managemental practices were also similar for all animals. Ten cows were considered for HS treatment, and similarly, ten cows were considered for TN treatment.

Samples from HS experimental animals were collected in the summer period (15 July to 30 August). In the summer season, THI was increased from 75 to 85 over one month and stabled at 80.50 for seven days. While the samples of TN treatments were gathered in the spring (15 march to 30 April). In the spring season, THI steadily increased from 58.8 to 62.3 over a one-month period. Before the onset of the experiment, all experimental animals were given an adaptation period of 15 days. A total of 30 days duration was given as an experimental period in the current study. All data were collected in 30 days of the experimental period.

Experimental animals were reared on total mixed ration (TMR). The frequency of feeding the animals was twice a day at 0500 and 1700. The TMR was supplied by the gate feeders, and 10% refusals were permitted. All experimental animals were milked twice a day (at 0500 and 1700). Animals were milked in a conventional milking parlor with a machine milking system (DeLaval, Tumba, Sweden). The milk production of each cow was recorded at each milking.

The diet used in this study was prepared to fulfill the requirements of a cow weighing 550 kg and producing 25 kg of milk (3.1% milkfat) daily, as suggested by National Research Council (NRC) 2001 [20]. Furthermore, the net energy for lactation (NEL) was 6.36 MJ/kg dry matter, which could meet the cow’s needs for TN conditions. The TMR’s ingredients and chemical composition are presented in Table 2.

### 2.2. Sampling and Analysis

The samples of fresh and refused TMR were collected on a weekly basis during the experimental duration (30 days). Collected samples of TMR were oven-dried at 65 °C for 24 h. Dried samples of TMR were ground through a 1 mm screen and were analyzed for dry matter, ash, calcium, phosphorus, Crude protein (CP), Neutral detergent fiber (NDF), and Acid detergent fiber (ADF). Proximate analysis was carried out following the protocol of Association of Official Analytical Chemists (AOAC) [21]. The contents of fiber in samples were determined following the protocol of Van Soest et al. [22].

In the last week of the summer period and spring period, morning milk samples (volume of 25 mL each) were collected at day 7 from the individual cow for the determination of milk components (fat, lactose, and protein) and somatic cells counts (SCC) (Arizona DHIA, Tempe, AZ, USA). In brief, a total of 25 mL of composite milk from each animal, with approximately equal volumes from each lactating udder quarter, was transferred to a sterile plastic bottle (Corning, Inc., Corning, NY, USA) and kept on ice until transport to the laboratory and storage at −80 °C for further analysis. Similarly, one-day milk samples (10 mL) from every cow were also collected on the last day of the experimental trial for ^1^H nuclear magnetic resonance (^1^H NMR) analysis. The collected sample of milk was defatted by centrifugation at 3000× *g* for fifteen min at four-degree Celsius. The skimmed milk samples were stored at −80 °C for H.NMR analysis.

Samples of blood were also collected from the coccygeal vein of the cows before morning feeding on the last day of the experimental trial. The blood samples were stored in evacuated tubes containing EDTA for anticoagulation. The collected samples were centrifuged, the same as described for the production of skimmed milk except for the centrifugation time. The centrifugation time was 10 min for blood samples. After the centrifugation procedure, blood plasma was aliquoted and stored at −80 °C until NMR analysis.

### 2.3. Measurements

Milk production and milk fat content were utilized to determine 4% fat-corrected milk. Rectal temperature (RTs) was determined 3 times per day by using a clinical thermometer (Nanjing, China). The respiratory rate was measured at 07:00, 14:00, and 22:00 twice a week by counting the total number of flank movements in one min and recorded as breaths per minute. DMI was recorded on a daily basis. DMI was measured by subtracting daily refusals from the daily offered feed. The following formula was used to calculate the temperature and humidity index (THI) following NRC 2001 [20].

THI = (Tdb + Twb) × 0.72 + 40.6(1)

where Tdb. = dry bulb temperature (°C), Twb. = wet bulb temperature (°C).

The Tdb and Twb temperatures were recorded daily at 0700, 1400, and 2200. Cows were weighed for 3 consecutive days with an empty stomach in the morning, both at the beginning and the end of the experiment.

### 2.4. ^1^H Nuclear Magnetic Resonance (^1^H NMR) Spectroscopic Measurement

We followed the procedure of sample preparation and NMR experiments, as described in the literature [23]. In brief, samples of blood plasma were melted at room temperature. Thawed plasma was homogenized by using a vortex mixer. After that, 400 µL of plasma sample was placed in a 1.5 mL plastic tube and mixed with 170 µL of D_2_O for detecting the signal. A 30 µL of Phosphate Buffer Saline (PBS) was added to reduce variations in the pH of the sample. After that, the samples of plasma were centrifuged at 12000 revolutions per minute (rpm) for ten minutes at four degrees Celsius. After centrifugation, 500 µL of supernatant was shifted into 5 Mm. nuclear magnetic resonance tubes. NMR tubes containing supernatant were stored at four degrees Celsius until further analysis. Nuclear magnetic resonance spectra of all samples were obtained at 298 K on a Bruker Avance 600 with a 599.91 Hz, the acquisition time of 0.9984 s, the spectral width of 8.01 kHz, and 2.1 s relaxation delay with 128 scans collected into 32K data points. One dimensional (1D) spectra were recorded using the Carr-Purcell-Meiboom-Gill (CPMG) experiment to suppress water signals and broad protein resonances.

All obtained ^1^H.NMR spectra were phased manually, and the baseline was adjusted by the use of MesReNova 7.1software. Before Fourier transformation, Free Induction Decay Signal (FIDs) were multiplied by an exponential with a 0.3Hz line broadening factor. The nuclear magnetic resonance spectrum was a reference to the lactate resonance at 1.33 ppm. Each spectrum (0.5–9.0 ppm) was split into 0.002 ppm bins, removing the residue water region from 5.2 to 4.5 ppm. The left-over bins of each spectrum were normalized to a total spectral area of unity prior to pattern recognition.

### 2.5. ^1^H NMR Data Handling

Free induction decays were multiplied by a factor of 1.0 Hz and phased manually. The chemical shift of each milk sample was referenced to the signal of the sodium trimethylsilyl (2, 2, 3, 3-2 H4) 1 propionate (TSP) (δ 0 ppm), and the chemical shift of each plasma sample was referenced to the signal of L-lactate (δ 1.33ppm). The NMR spectra of milk samples (δ 0.6–9.0 ppm) and plasma samples (δ 0.5–9.0 ppm) were further divided into 0.002 ppm integral region and integrated. The parts of δ 4.73–5.16 ppm and δ 3.33–3.34 ppm were eliminated to eradicate the baseline effect of imperfect water supersession and effects of ethanol on milk samples, and the region of δ 4.5–5.2 ppm was removed to eradicate baseline effects of inadequate water supersession on plasma samples.

Multivariate analyses, comprising Principal Component Analysis (PCA) and Orthogonal Partial Least Squares Discrimination Analysis (OPLS-DA), were carried out by the use of SIMCA-P11.0 software (UmetricsUmea, Sweden). PCA is known to show the core structure of datasets in an unbiased pattern and to reduce the dimensionality of experimental data. In the current experiment, PCA was used to explore the global clustering and variations in metabolic profiles between obtained samples. Furthermore, the data of mean center scaling was employed for PCA. For regression, partial least square discrimination analysis was used, and the R^2^X, R^2^Y, and Q^2^ parameters were carried out to assess the model quality. The R^2^Y and R^2^X parameters, which characterize the fractions of X and Y variations, respectively, were utilized to assess the quality of the model. The predictive ability of the model was represented by the Q^2^ parameter. Usually, the model is acceptable when the values of R^2^Y, R^2^X, and Q^2^ are more significant than 0.5. After obtaining the initial overview of PCA and Partial Least Squares Discrimination Analysis (PLS-DA) analysis, a more sophisticated OPLS-DA model with the precise discriminant info was obtained from the HS and TN periods.

The difference of the metabolites in experimental treatments is represented as a coefficient of variation plots. The OPLS-DA model was utilized to increase the separation between samples by eliminating the variation in the X matrix unrelated to the Y matrix [24]. The multivariate models were validated by a 6-round cross-validation procedure to guard against overfitting and by permutation tests (*n* = 200). The color-coded coefficient of loading plots of the OPLS-DA model was utilized to determine the difference between the HS and TN periods.

Based on the number of samples in the current experiment, the correlation coefficients of |r| > 0.63 and |r| > 0.60 were used as the cutoff values for the significance of milk and plasma samples, respectively.

### 2.6. The Differential Metabolites Identification and the Analysis of the Metabolic Pathway

The OPLS-DA and PLS-DA were used to identify the difference in metabolites. SPSS 16.0 was used to calculate the receiver operating characteristic (ROC) curves, and the diagnostic value of these differentials was determined by the areas under the curves (AUCs). The Kyoto Encyclopedia of Genes and Genomes (KEGG) was used to find the metabolic pathways and enrichment analysis. After that, MetaboAnalyst3.0 (https://www.metaboanalyst.ca/) was used for the pathway analysis.

### 2.7. Calculation and Statistical Analysis

The effects of HS on physiological parameters and milk yield were analyzed by repeated-measures by using the PROC GLM procedure of SAS 9.0 software (SAS Institute Inc., Cary, NC, USA). Least square means were separated by using the Pare difference (PDIFF) procedure of SAS 9.0 software (SAS Institute Inc, Cary, NC, USA). The significance was declared at *p* < 0.05.

## 3. Results

### 3.1. Environmental Conditions

**Figure 1:**
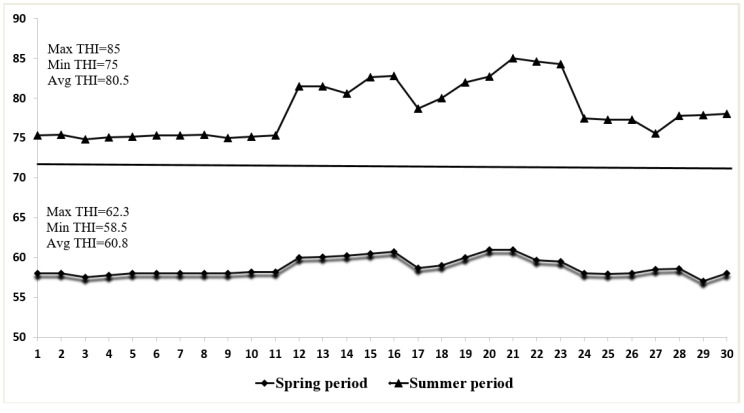
The temperature and humidity index (THI) values (mean/d) during the summer period (*n* = 10, triangle line) and spring period (*n* = 10, rectangle line).

The THI values calculated during the experimental periods are shown in Figure 1. Results revealed that daily mean THI was between 58.5 and 62.3 during the spring season (avg. 60.8 in the whole spring season). The daily mean THI was ranged from 75.0 to 85.0 during the summer season (avg. 80.5 in the whole summer season). The mean daily THI in the entire summer period exceeded 72, indicating that dairy cows in the HS treatment suffered HS.

### 3.2. Physiological Parameters and Animal Performance

The physiological parameters and performance of cows are illustrated in Table 3. Bodyweight did not differ both at the beginning and the end of the HS and TN periods. However, body weight change in the experimental treatments varied greatly (*p* < 0.01). The respiratory rates and RTs were higher (*p* < 0.01) in the HS experimental treatment than the TN treatment.

Heat stress reduced milk production (*p* < 0.05), milk protein contents (*p* < 0.05), milk protein yield (*p* < 0.01), and DMI (*p* < 0.05). Heat stress did not alter milk lactose contents and milk fat contents (*p* > 0.10). Results showed that milk fat yield and lactose yield were lowered in the HS experimental cows as compared to the TN experimental group animals. Results showed that HS increased the SCC (*p* = 0.03) of cows.

### 3.3. ^1^H NMR Spectra of Milk and Blood Plasma Samples

The representative 600 MHz of Varian 600 (Agilent, Santa Clara, CA, USA) spectra gave an overview of the metabolic profiles from milk and plasma (Supplementary Materials Figures S1–S3) of cows in the HS and TN groups. By visual inspection of the ^1^H NMR spectra, different metabolite patterns were observed between the experimental groups. Milk concentrations of pyridoxamine and scyllo-inositol and plasma concentrations of glucose and formate appeared to be higher in cows of the HS group. To get a more intensive analysis of the difference in metabolites in the experimental treatments, NMR data was further analyzed by using multivariate analysis, including PLS-DA, PCA, and OPLS-DA, to obtain the significant differences of obtained metabolites between experimental treatments.

### 3.4. Identification of Different Metabolites

**Figure 2:**
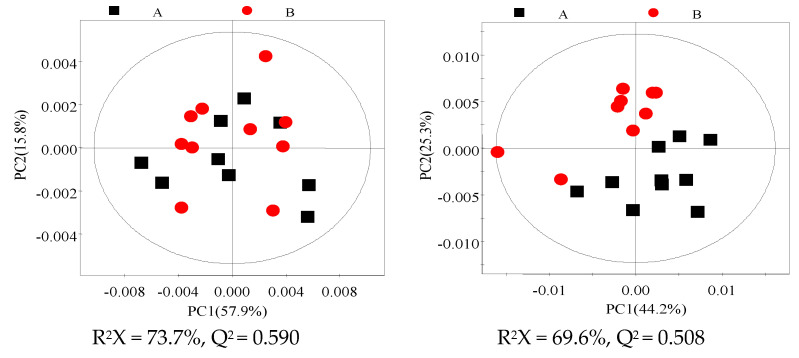
Principal Component Analysis (PCA) scores plot based on ^1^H.NMR spectrum of aqueous phase acquired from the HS (heat stress) and TN treatments (**left**). PCA scores plot based on ^1^H NMR spectra of plasma acquired from the HS and TN treatments (**right**). A = heat stress (HS) group; B = thermal neutral (TN) group.

**Figure 3:**
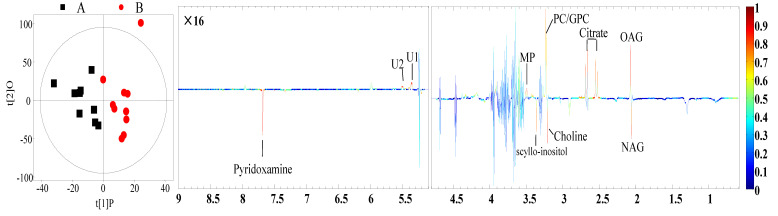
Orthogonal Partial Least Squares Discrimination Analysis (OPLS-DA) scores plots (**left panel**) and the corresponding coefficient loading plots (**right panel**) of milk. A = heat stress treatment; B = thermal neutral treatment.

**Figure 4:**
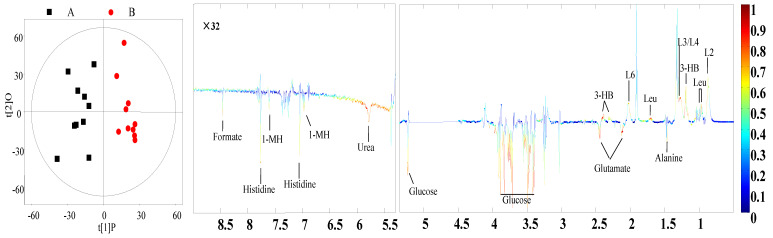
OPLS-DA scores plots (**left panel**) derived from the ^1^H. NMR spectra of plasma and the corresponding coefficient loading plots (**right panel**) acquired from the different pairwise groups. A = heat stress group; B = thermal neutral group.

**Figure 5:**
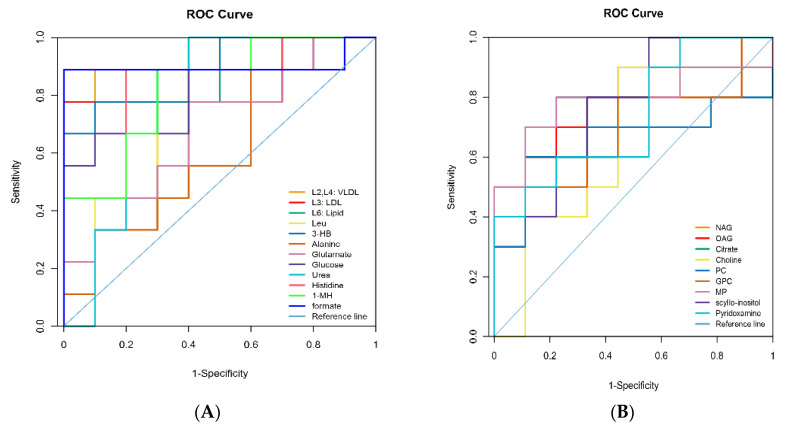
ROC (receiver operating characteristics) analysis of biomarkers from the milk (**A**) and plasma (**B**) for cows from the HS (heat stress) and TN (thermoneutral) periods.

The PCA and PLS-DA of the ^1^H.NMR data from milk and plasma represented a separation in the experimental treatments (Figure 2, Supplementary Materials Figures S4 and S5). OPLS-DA analysis revealed that concentrations of eight metabolites in the samples of milk and twelve metabolites in the samples of plasma were significantly different between the HS and TN experimental groups (Figure 3 and Figure 4). The cows in the HS groups had a higher level of choline, N-acetyl glycoprotein (NAG), scyllo-inositol, and pyridoxamine and lower levels of citrate, O-acetyl glycoprotein (OAG), glycerol phosphorylcholine (GPC), and methyl phosphate in milk as compared to cows in the TN groups. The cows from the HS group had greater levels of glutamate, alanine, glucose, histidine, urea, 1-methylhistidin, and formate and lower levels of Very low density lipoprotein (VLDL), low density lipoprotein (LDL), lipid, leucine, and 3-hydroxybutyrate in plasma as compared to the cows in the TN experimental group (Table 4). We further employed ROC analysis to find the diagnostic values of obtained different metabolites for distinguishing the HS experimental cows from the TN experimental cows (Figure 5A,B). Most of the metabolites showed excellent diagnostic potential with the AUC values of 0.70–1.0.

### 3.5. Integration of Key Different Metabolic Pathways

**Figure 6:**
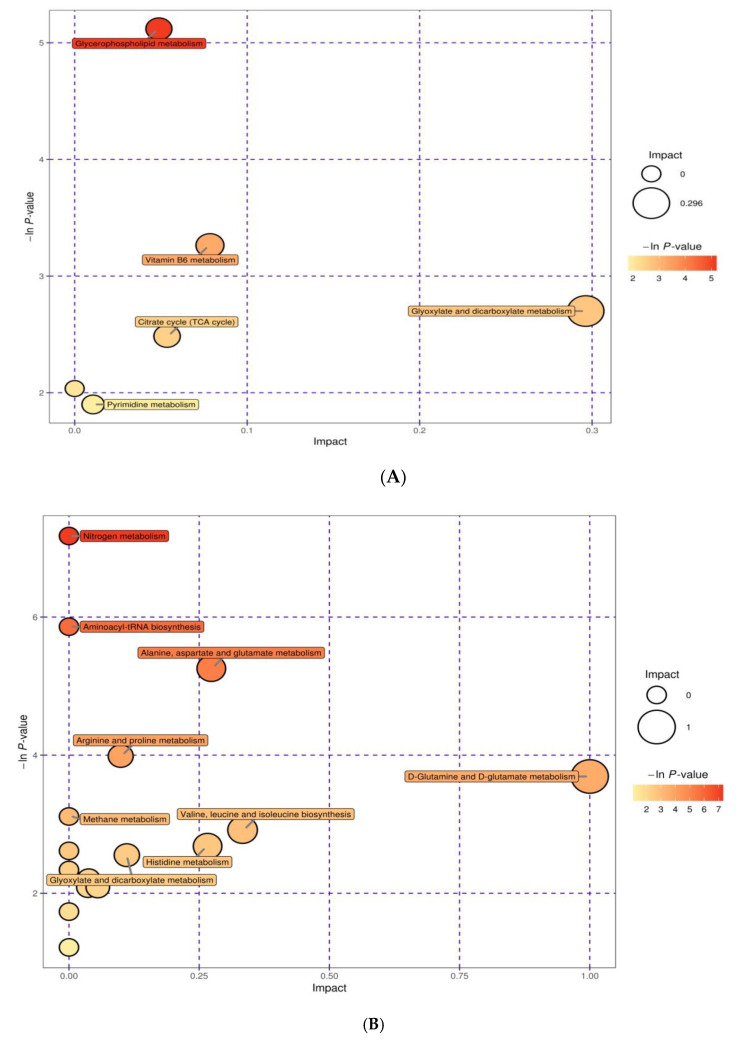
The metabolome view map of significant metabolic pathways characterized in milk (**A**) and plasma (**B**) for cows from the HS (heat stress) and TN (thermoneutral) periods. This figure tries to find pathways that were significantly changed based on enrichment and topology analysis.

**Figure 7:**
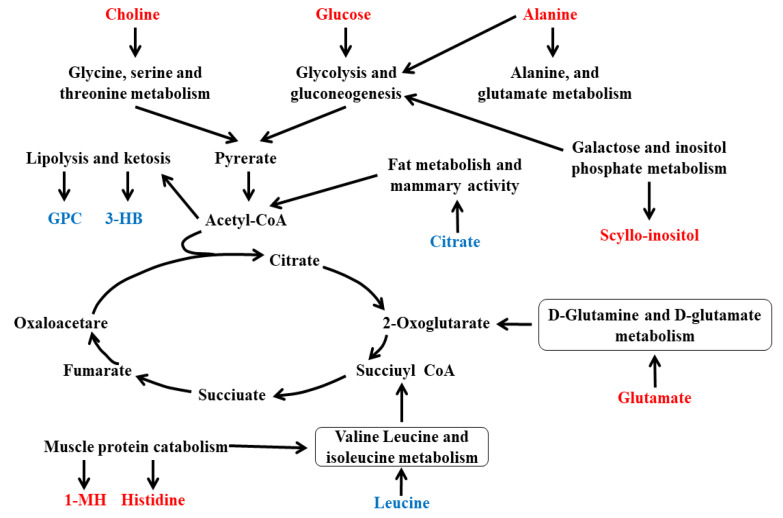
Metabolic network of the potential biomarkers in plasma and milk, which were different between the HS (heat stress) and TN (thermoneutral) periods. The increased metabolites in the HS period are represented in red. The decreased metabolites in the HS period are represented in blue. GPC: glycerol phosphoryl choline; 3-HB: 3-hydroxybutyrate; 1-MH: 1-Methylhistidine.

In the current experiment, a total of twelve pathways were obtained. Those metabolites were involved in various biochemical pathways, such as proteolysis, glycolysis, lipolysis, gluconeogenesis, galactose, inositol phosphate metabolism, muscle protein catabolism, purine metabolism, and alanine metabolism (Figure 6A,B). The obtained results were combined to draw a metabolic network map, as shown in Figure 7, to represent the biological significance of the variation of plasma and milk concentrations of metabolites as a result of HS.

## 4. Discussion

In dairy production systems, THI has been used as a vibrant indicator to evaluate HS in dairy cows. It is normally believed that THI above 68 results in HS in dairy cows [25]. In the present experiment, THI results showed that average THI was above 80.5 in the HS experimental group (Figure 1), indicating HS in cows of this group. Moreover, previous studies have reported that higher RTs and respiration rates are prominent indicators of heat-stressed dairy cows [26,27,28,29]. In the present experiment, higher RTs and respiration rates in cows of the HS experimental treatments further clarified that cows in the HS experimental treatment suffered from HS.

In this study, HS reduced DMI and milk production, which is similar to the findings of previous studies [1,7,30]. It has already been reported that HS reduces the DMI, milk yield, and milk protein contents [15,19]. However, recent studies have explored that in heat-stressed dairy cows, low DMI only partially describes the dairy cow milk productivity and changes in milk composition [17,18]. In the current study, HS reduced DMI dramatically in cows, and thus cows were unable to meet energy demands for maintenance and lactation. Therefore, it could be assumed that HS cows were in negative nutrient balance, as supported by bodyweight changes in HS cows as compared to cows in the thermoneutral zone (Table 3). In negative energy balance, processes of both lipolysis and proteolysis started in cows [31].

3-Methylhistidine (3-MH) is a product of the posttranslational methylation of histidine in both myosin and actin, and the plasma concentration of 3-MH is a good indicator of protein mobilization in cows because it is not further metabolized in the body [32]. Interestingly, we did not find the changes in the plasma concentration of 3-MH. Instead, we found the upregulation of 3-Methylhistidine(1-MH)in the plasma of heat-stressed dairy cows. 1-MH is the degradation product of anserine (β-alany1-1-methy1-histidine) that is a dipeptide found in the muscles of many animals, including cows [33]. 1-MH is used as a biomarker for meat intake in humans because anserine is not found in human muscle [34]. This methylation product of histidine cannot be synthesized in the human body unless anserine-containing meat is consumed. In the current study, it could be assumed that anserine was degraded in cow muscle and resulted in the production of 1-MH during HS, which ultimately enhanced the plasma concentration of 1-MH. Therefore, the plasma concentration of 1-MH could be a possible biomarker for monitoring protein mobilization in HS dairy cows.

In normal and pathological conditions, the rate of protein synthesis is correlated with the intracellular amino acid pool as compared to the blood plasma concentration of amino acid [35,36]. Results showed that HS increased concentrations of glutamate, alanine, and histidine in the plasma of cows. It has been reported that glutamate, alanine, and histidine are the main precursors of glucose production via gluconeogenesis [12,37]. It has also been reported that during periods of food deprivation, gluconeogenesis and glycolysis are regulated by alanine to ensure glucose production [37,38]. Furthermore, it has also been reported that under HS condition, alanine synthesis increases, and alanine accumulates as the major amino acid [16,39]. In the current study, higher levels of alanine were found in the HS experimental group, representing that during HS, gluconeogenesis was started in cows. Therefore, high plasma alanine concentration is also a sign of inadequate cellular energy substrates of dairy cows in HS periods.

Glutamate is essential for carbohydrate and amino acid metabolism because it is a chief precursor of glucose synthesis during the process of gluconeogenesis [40]. It has been reported that in many amino acids’ metabolism, proline, ornithine, glutamine, arginine, and histidine converge on glutamate itself [41]. Moreover, under stressful cell conditions, glutamate can be synthesized for neurotransmitters and glutathione to protect cells from damage from the redox crisis [42]. It has been reported that HS increases the synthesis of glutamate, which is often part of an adaptive strategy for the body in response to stressors [12,16,37,39]. A higher level of glutamate was found in the heat-stressed cows, highlighting that glutamate was involved in multiple metabolic processes under HS. The accumulation of excess amino acids in the plasma indicated that amino acid catabolism was probably started in the liver [43]. These amino acids can further degrade to produce 2-oxoglutarate. Through the tricarboxylic acid cycle (TCA), 2-oxoglutarate is converted to oxaloacetic acid, which is used to synthesize glucose to supplement the energy demand. Based on findings, it could be concluded that dairy cows subjected to HS suffer from protein catabolism and lack of energy substrates.

Formate is an essential endogenous one-carbon metabolite in animals, who participate in a vital one-carbon pool of intermediary metabolism, and used as a valuable biomarker for impaired one-carbon metabolism [44]. In dairy production, the endogenous source of formate is rumen methanogens, which use microbial fermentation to produce products, such as formic acid and hydrogen, which are reduced to produce methane. Therefore, there is normally little hydrogen and formic acid in the rumen [45]. Our results showed that plasma concentrations of formate in heat-stressed cows were increased, suggesting that HS led to impaired methanogenesis, which led to high levels of formate entering the blood. Formate is observed to be toxic to animals as it has been reported to be involved in the toxicity observed with methanol poisoning [46]. Metabolic acidosis and optic nerve damage are connected with the toxicity of formate [47]. Hovda et al. [48] demonstrated that metabolic acidosis happened only at higher concentrations of plasma formate. Our results showed impaired one-carbon metabolism and possible signs of a metabolic acidosis of the cows during the HS period.

Current study findings explored that plasma concentrations of glucose were increased in the animals of the HS group, similar to the research results of Srikandakumar and Johnson in Holstein cows [49]. The higher plasma concentration of glucose could be explained by the theory of higher uptake of glucose from the kidney and the intestine and the higher production of hepatic glucose during HS [50,51,52].

Significantly, decreased levels of 3-hydroxybutyrate (3-HB) were observed in the HS animals. The 3-HB, the main ketone body, is generated from lipolysis in the liver mitochondria and could be utilized as an energy source as the supply of blood glucose is insufficient [53]. Therefore, the decreased 3-hydroxybutyrate plasma levels in heat-stressed cows might indicate that the inhibition of β-oxidation of fatty acid occurred in cows suffering HS [54]. The upregulation of plasma concentrations of glucose in the HS period in this study supported the theory that β-oxidation of fatty acid is inhibited in heat-stressed cows. Previous studies have also reported that HS limits fat mobilization to limit heat generation [55,56]; therefore, it could be assumed that lipolysis may not be an energy-generating process for dairy cows during HS. However, further evidence is required to confirm this hypothesis. In addition, plasma 3-HB is derived not only from liver fatty acid metabolism but also from rumen butyrate [57]. A decrease of DMI in animals of the HS experimental group may be related to a decrease in plasma 3-hydroxybutyrate. In any case, the decreased plasma 3-HB in this study might suggest the inhibitory effect of HS on lipolysis in dairy cows. Plasma 3-HB level may also be used as a biomarker for evaluating the susceptibility to HS.

A significantly decreased level of leucine was seen in cows of the HS experimental groups in the current study. The essential amino acid leucine can stimulate muscle protein synthesis [56]. Fried et al. [58] stated that the increased concentration of leucine in the blood led to an increase in protein turnover in a mouse model. A decreased leucine concentration in blood plasma causes physical and mental fatigue [59]. Leucine is also concerned with different disease conditions; for example, the disease of maple syrup urine disease [60]. It has also been reported that the metabolism of leucine is essential for heart disease [61]. Taegtmeyer et al. [62] observed that the supplementation of leucine aided in repair of the heart during ischemic injury. Therefore, it could be hypothesized that the decreased level of leucine observed in our study might lead to mental fatigue and ischemic injury of the heart of dairy cows during HS.

VLDL is likely an essential source of fats for extrahepatic tissues [63]. Our results showed that the plasma concentration of VLDL decreased due to HS. The result of the lower plasma level of VLDL is similar to the research results of Tian et al. [16], who observed that HS negatively influenced the level of plasma VLDL in the animals suffered from HS. It is known that hepatocytes are involved in the assembly and secretion of VLDL particles [64]. The lower level of VLDL could be justified with the lesser production of the essential components of VLDL like cholesterol, apolipoproteins, and phospholipids (especially GPC) [65]. In the current study, the level of GPC decreased, but the concentration of choline was upgraded in the milk of HS cows. This result might indicate that HS causes abnormalities in liver lipid metabolism. It has been reported that a reduced ratio of GPC to choline leads to a metabolic change of phosphatidylcholine and GPC to choline during the HS period [66]. It has been further reported that decreased GPC levels in the milk are advantageous for choline release and adaptation to hot weather conditions. Previous studies have also reported that choline is positively associated with good coagulation parameters [67]. The high choline content, together with the decreased citrate content in the dairy cow, during the HS period, could improve milk coagulation properties [67].

As a common protein modification, protein glycosylation is linked to protein functions, such as biological recognition, enzymatic protection, and protein folding [68,69]. The N-acetyllactosamine units are typically present in glycoprotein, which is constituted of an O- or N-linked core structure. It has been reported that N-acetyllactosamine core structures are carriers of terminal carbohydrate epitopes, which confer a specific biological property to the glycoprotein [69]. Furthermore, it is believed that approximately all of the important molecules concerned with the adaptive and innate responses are glycoproteins, and the majority of circulating glycoproteins are acute phase reactants and immunologic proteins. Many studies have reported that HS impairs cow immune function [70,71] and results in higher disease incidence, especially in the period after postpartum [72]. Moreover, it has also been reported that HS also has a negative impact on the offspring [73]. The study of Nardone et al. [74] explained that HS in dairy cows during the late pregnancy reduced the concentration of IgA and IgG and the percentage of total protein of the colostrum from the first milking of primiparous dairy cows. In another study, higher mortality in heat-stressed neonatal calf was observed due to impaired colostral antibody absorption [75]. So far, no study has reported any glycan-dependent functions for the glycoproteins present in the cow’s milk. Therefore, in the current experiment, the increased milk level of NAG and the decreased milk level of OAG in animals of the HS experimental groups might have experienced an impaired immune function and incorrect glycosylation of immunoglobulins by mammary gland epithelial cells. Thus, both NAG and OAG may be useful milk biomarkers for heat-stressed cows.

Scyllo-inositol is an isomer of myoinositol, which can be produced in vivo from glucose-six-phosphate [76]. Inositol is a galactose metabolism product, and it has been reported that a part of serum inositol is derived from the degradation of dietary phytate by some specific species of intestinal microbes [77]. Milk scyllo-inositol may either be up taken from blood or synthesized by mammary gland epithelial cells. It has been reported that in transgenic mice with an Alzheimer’s phenotype, scyllo-Inositol reverses the memory deficit [78]. In the current study, the content of scyllo-inositol was upregulated by HS, which indicated that HS leads to inositol metabolism disorder and cellular toxicity of mammary gland epithelial cells.

Pyridoxamine is one form of vitamin B6, serving as a coenzyme in various enzymatic reactions, such as transamination and decarboxylation reactions [79]. The deficiency of vitamin B6 can cause various processes of the body, such as nephrotic syndrome and inflammation [80]. Milk pyridoxamine, therefore, is an essential functional nutrient beneficial to animal health. However, the biological significance and the mechanism of the elevated milk concentration of pyridoxamine during the HS period is not known.

Citrate is not the main constituent of milk, but it plays as a buffer role in the udder of dairy cows. It has been reported that citrate regulates Ca^2+^and H^+^ ions homeostasis and maintain the fluidity of milk by effecting casein micelles [81]. In the case of citrate deficiency in the udder, the clumping of Ca^2+^ appears, which causes injury to the parenchymatous tissues of the alveoli of the udder and leads to the damage of barriers between blood and milk and inflammatory reactions in the alveolar tissue of the udder [82]. In the present experiment, the level of citrate in milk decreased significantly during the HS period, indicating that HS may seriously affect udder health. Furthermore, citrate is known to be involved in the TCA, and in dairy cows, it is known as a biomarker of energy balance [83]. Moreover, citrate is correlated with ketone bodies in milk and de novo fatty acids synthesis [84]. There is strong evidence that citrate in milk comes from citrate produced within the mammary secretory cell rather than from that in the blood plasma [85]. The previous study of Tian et al. [16] reported that the HS decreased the milk citrate concentration due to changes in mammary gland function rather than a disturbance in the blood citrate metabolism. Therefore, the lower level of citrate in heat-stressed cow’s milk could reflect the decreased mammary activity rather than imbalanced energy metabolism. In the current experiment, HS increased the SCC, similar to the findings of the recent study [86]. It has been reported that higher milk SCC due to HS indicates a mammary immune response to simulated infection [87]; therefore, SCC results of the current study suggest that HS may seriously affect udder health.

## 5. Conclusions

In this study, results explored that HS reduced the DMI and influenced the metabolites in the milk and plasma of dairy cows. Results showed that a total of eight metabolites in milk and 12 metabolites in plasma were altered due to HS. These metabolites were mainly involved in gluconeogenesis, protein degradation and synthesis, and milk fat synthesis. These metabolites in the milk and plasma could be potential biomarkers for HS. In addition, this work found several metabolites (especially in milk) that have rarely been studied.

## Figures and Tables

**Table 1:** Characteristics of the experimental cows.

Parameter	TN ^1^ (Thermal Neutral)	HS ^2^ (Heat Stress)	*p*-Value
Number of cows	10	10	
Parity	3.0 ± 1.0	3.2 ± 1.1	0.78
Lactation days	146.2 ± 12	145 ± 25	0.95
Milk yield (kg)	26.8 ± 1.5	27.2 ± 2.1	0.95
Average bodyweight (kg)	604.4 ± 56.44	601.81 ± 78.97	0.95

^1^ TN: Thermal neutral. ^2^ HS: heat stress.

**Table 2:** Composition (%. of dry matter (DM)) and nutritional value of the diet.

Item	%
Ingredient	
Silage (Corn silage)	19.63
Alfalfa hay	5.82
*Hemarthriia altissima* ^1^	10.60
Sweet potato vine	13.95
Corn meal	23.50
Wheat bran	6.00
Soybean meal	9.70
Rapeseed meal	6.00
Calcium fatty acids	1.00
Limestone	1.20
Calcium superphosphate	1.00
Salt	0.60
Premix ^2^	1.00
Total	100
Roughage-to-concentrate	50/50
Nutrient levels ^3^	
NEL ^4^ (MJ/kg)	6.36
NEL (Mcal/kg)	1.51
CP ^5^	16.18
NDF ^6^	36.46
ADF ^7^	22.96
Ca ^8^	1.06
*P* ^9^	0.56

^1^*Hemarthria altissima* and sweet potato vine fresh mowing. ^2^ Premix contained (per kilogram of premix): VA— 5,00,000 IU, VD— 1,50,000 IU, VE—3000 IU, Fe (iron) —4.0 g, Cu (copper) —1.3 g, Mn—3.0 g, Zn (Zinc)—6.0 g, I (iodine)—80 mg, Se (selenium)—50 mg, and Co (cobalt)—80 mg. ^3^ Net energy for lactation was the calculated value; the others were measured values. ^4^ NEL: Net energy for lactation; ^5^ CP: Crude protein; ^6^ NDF: Neutral detergent fiber; ^7^ ADF: Acid detergent fiber; ^8^ Ca: Calcium; ^9^ P: Phosphorus.

**Table 3:** Physiological parameters and performance of cows reared in heat stress and thermoneutral periods (mean ± SD).

Item	Thermoneutral Period	Heat Stress Period	*p*-Value
Respiration rate (breaths/min)	35.4 ± 4.7	82.3 ± 10.5	<0.01
Rectal temperature (°C)	38.6 ± 0.1	39.4 ± 0.3	<0.01
Initial ^1^ BW (kg)	604.4 ± 56.4	601.8 ± 79.0	0.91
Final BW (kg)	612.2 ± 55.6	565.5 ± 74.6	0.17
BW changes (kg)	7.8 ± 8.3	−36.4 ± 11.2	<0.01
^2^ DMI (kg)	18.1 ± 2.5	15.5 ± 2.0	<0.05
Milk yield (kg/d)	28.6 ± 1.4	19.9 ± 2.1	<0.05
Protein (%)	3.2 ± 0.2	2.9 ± 0.9	<0.05
Protein yield (g/d)	914.5 ± 2.2	578.1 ± 1.8	<0.01
Fat (%)	3.6 ± 0.7	3.8 ± 0.6	0.12
Fat yield (g/d)	1026.6 ± 3.2	753.8 ± 2.5	<0.05
Lactose (%)	4.9 ± 0.2	4.7 ± 0.2	0.19
Lactose yield(g/d)	1407.2 ± 5.2	933.5 ± 3.8	<0.05
Somatic cells count 1000/mL	286± 8.2	312± 9.5	0.03

^1^ BW = bodyweight, ^2^ DMI = dry matter intake.

**Table 4:** OPLS-DA coefficients derived from the ^1^H NMR data of metabolites in the milk and plasma of cows reared in the heat stress and thermoneutral zone.

	Metabolites ^1^	Identification (ppm) and Multiplicity ^2^	Correlation Coefficients (r) ^3^	*p*-Value
Milk	N-Acetyl glycoprotein	2.06 (s) ^4^	−0.73	0.02
	O-Acetyl glycoprotein	2.07 (s)	0.68	0.01
	Citrate	2.53 (d), 2.67 (d) ^5^	0.74	<0.01
	Choline	3.20 (s)	−0.64	0.03
	Glycerophosphorylcholine	3.23 (s)	0.63	0.01
	Methyl phosphate	3.49 (d)	0.68	0.1
	scyllo-Inositol	3.36 (s)	−0.66	0.02
	Pyridoxamine	7.67 (s)	−0.68	0.02
Plasma	L2, L4: VLDL	0.89 (br), 1.29 (br) ^6^	0.81	<0.01
	L3: LDL	1.27 (br)	0.66	<0.01
	L6: Lipid	2.01 (br)	0.64	<0.01
	Leucine	0.96 (t) ^7^, 1.69 (m) ^8^	0.75	<0.01
	3-Hydroxybutyrate	1.20 (d)	0.74	<0.01
	Alanine	1.48 (d)	−0.64	<0.01
	Glutamate	2.13 (m), 2.46 (m)	−0.65	<0.01
	Glucose	3.42 (t), 3.54 (dd) 3.71 (t), 3.73 (m) 3.84 (m), 5.23 (d)	−0.71	<0.01
	Urea	5.78 (br)	−0.66	0.02
	Histidine	7.05 (s), 7.76(s)	−0.63	<0.01
	1-Methylhistidine	6.96 (s), 7.61(s)	−7.64	<0.01
	Formate	8.45 (s)	−0.68	<0.01

^1^ VLDL: very-low-density lipoprotein; LDL, low-density lipoprotein. ^2^ Multiplicity: s represents singlet, d represents doublet, t represents triplet, q represents quartet, dd represents doublet of doublets, m represents multiplet, br represents broad resonance. ^3^ Correlation coefficients: positive and negative signs show a positive and negative correlation in the concentrations of milk metabolites in dairy cows in the TN (thermoneutral) group relative to cows in the HS (heat stress) group. ^4^ s: singlet; ^5^ d: doublet; ^6^ br: broadlet; ^7^ t: triplet; ^8^ m: multiplet.

## Supplementary Materials

The following supplementary material is available online at https://www.mdpi.com/2076-2615/10/10/1741/s1. Figure S1: A representative 600 MHz ^1^H.NMR spectrum (δ0.5–5.0 and δ5.0–9.5) of milk from the HS period and TN period, Figure S2: A representative 600 MHz ^1^H.NMR spectrum (δ0.5–5.5 and δ5.5–9.0) of plasma from the HS period and TN period, Figure S3: least squares discrimination analysis (PLS-DA) scores plots (**a**) derived from ^1^H.NMR spectra of the aqueous phase of milk extracts. A = heat stress group; B = thermal neutral group, Figure S4: PLS-DA scores plots (**a**) derived from ^1^H.NMR spectra of serum obtained from different groups, and cross-validation (**b**) by permutation test (*n* = 300). A = heat stress group; B = thermal neutral group. Figure S5: Partial least squares discrimination analysis PLS-DA score plots (left panel) derived from ^1^H NMR spectra of serum obtained from different groups and cross validation (left panel) by permutation test (*n* = 300). A = Heat Stress group; B = thermal neutral group.
