# Supplementary material for: Metabolomics Approach Explore Diagnostic Biomarkers and Metabolic Changes in Heat-Stressed Dairy Cows
Source: Animals (Basel). 2020 Sep 25;10(10):1741. doi: 10.3390/ani10101741 (PMC7601318; doi:10.3390/ani10101741)
Supplement: Supplementary file 1 [file animals-10-01741-s001.pdf]

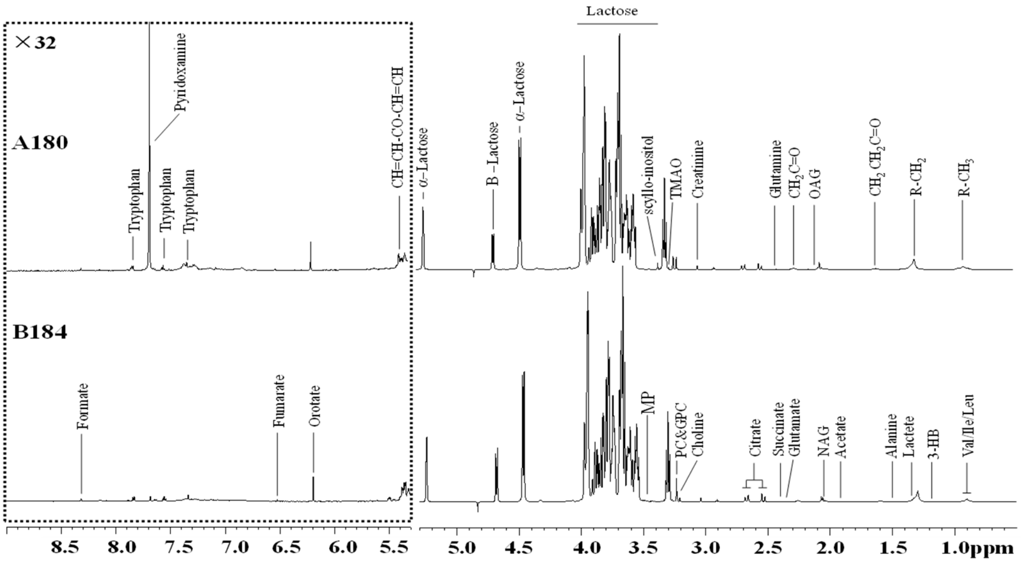

Figure S1. A representative 600 MHz 1H-NMR spectrum (δ0.5–5.0 and δ5.0–9.5) of milk from the HS period and TN period. The region of δ5.3–9.5 (in the dashed box) was magnified 32 times compared with the corresponding region of δ0.5–5.3 for the purpose of clarity.A = Heat Stress group; B = thermal neutral group. 1-MH: 1-Methylhistidine; 3-HB: 3-Hydroxybutyrate; Crea: creatine; Glu: glutamate; GPC: glycerophosphorylcholine; Ile: isoleucine; L1 and L3 lipid: LDL; L2, L4, and L5 lipid: VLDL; L6 lipid: –CH2–CH=CH–; L7 lipid: –CH2–CH=CH–; L8 lipid: –CH2–C=O; L9 lipid: =CH–CH2–CH=; Leu: leucine; Lys: lysine; NAG: N-acetyl glycoprotein; PC: phosphocholine; Phe: phenylalanine; Val: valine.

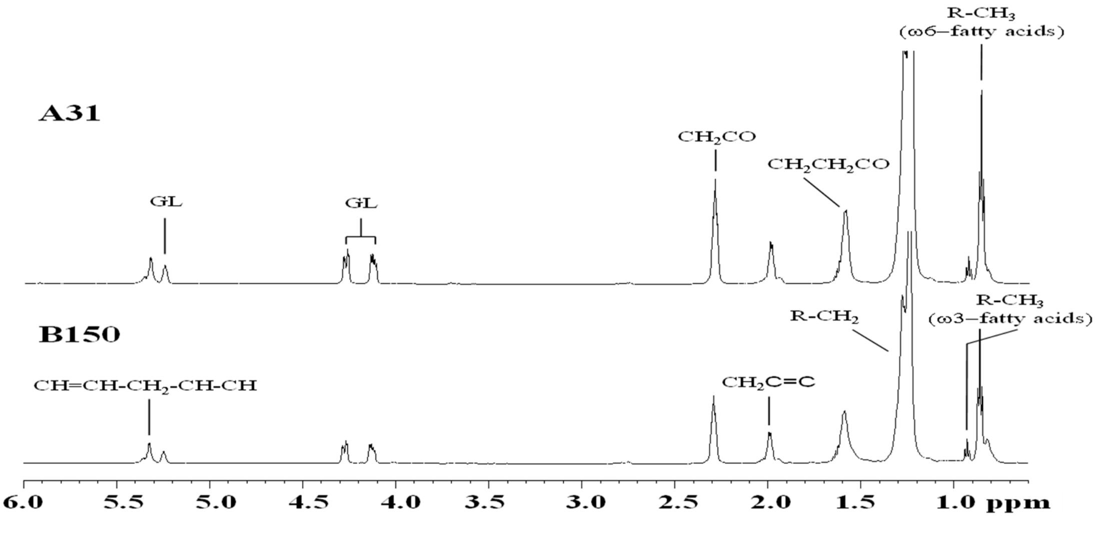

Figure S2. 600 MHz ^{1}H NMR spectra (δ0.5-6.0) of lipid phase of milk extracts from groups A318 and B150.A = Heat Stress group; B = thermal neutral group. GL: Glyceryl of lipid.

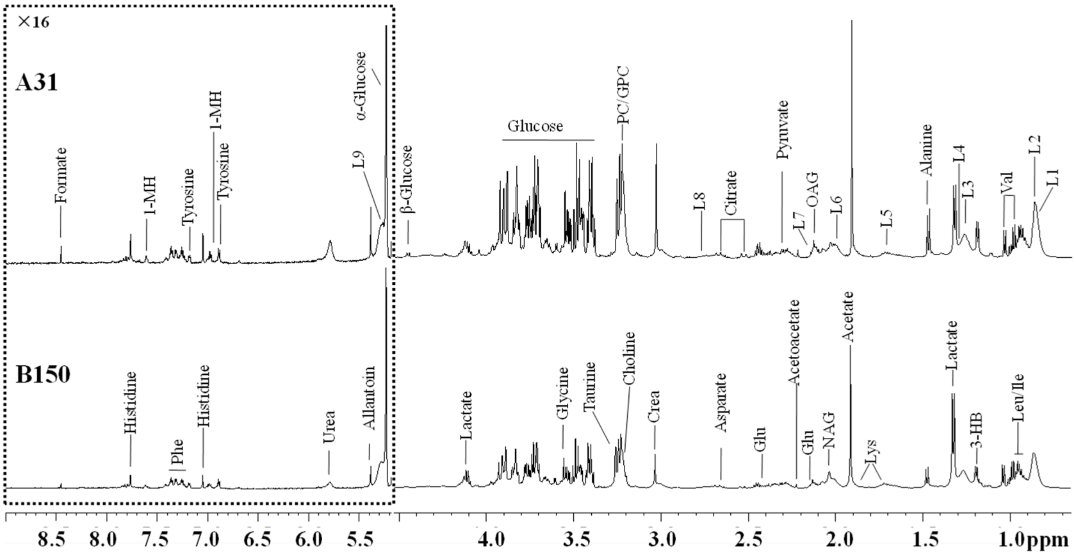

Figure S3. A representative 600 MHz ^1H NMR spectrum (δ0.5–5.5 and δ5.5–9.0) of plasma from the HS period and TN period. The region of δ5.2–9.0 (in the dashed box) was magnified 16 times compared with the corresponding region of δ0.5–4.6 for the purpose of clarity.A = Heat Stress group; B = Thermal neutral group. 3-HB: 3-Hydroxybutyrate; GPC: glycerophosphorylcholine; Ile: isoleucine; Leu: leucine; MP: methyl phosphate; NAG: N-acetyl glycoprotein; OAG: O-acetyl glycoprotein; PC: phosphorylcholine; TMAO: trimethylamine N-oxide; Val: valine.

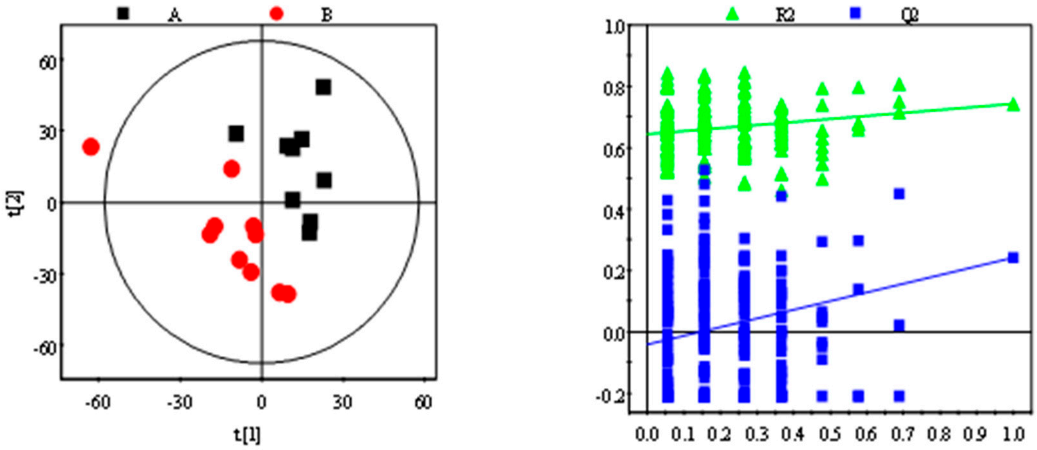

$R^2X = 43.7\%$, $R^2Y = 74.3\%$, $Q^2 = 0.242$

Figure S4. Partial least squares discrimination analysis (PLS-DA) score plots (left panel) derived from ^1H NMR spectra of the aqueous phase of milk extracts. A = Heat Stress group; B = Thermal neutral group.

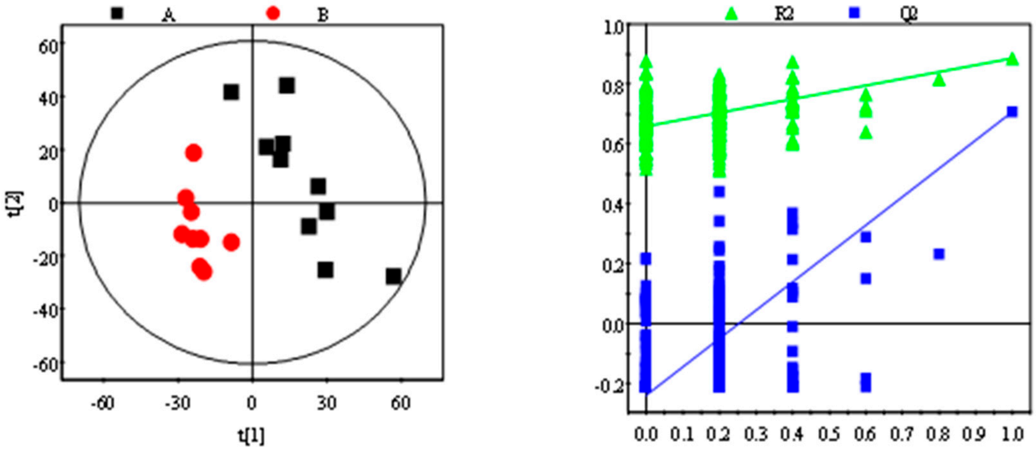

$R^2X = 34.6\%$, $R^2Y = 90.8\%$, $Q^2 = 0.641$

Figure S5. Partial least squares discrimination analysis PLS-DA score plots (left panel) derived from ^{1}H NMR spectra of serum obtained from different groups and cross validation (left panel) by permutation test (n = 300). A = Heat Stress group; B = thermal neutral group.
